# Neurotrauma Surveillance in National Registries of Low- and Middle-Income Countries: A Scoping Review and Comparative Analysis of Data Dictionaries

**DOI:** 10.34172/ijhpm.2021.167

**Published:** 2021-12-06

**Authors:** Ernest J. Barthélemy, Anna E. C. Hackenberg, Jacob Lepard, Joanna Ashby, Rebecca B. Baron, Ella Cohen, Jacquelyn Corley, Kee B. Park

**Affiliations:** ^1^Program in Global Surgery and Social Change, Department of Global Health and Social Medicine, Harvard Medical School, Boston, MA, USA.; ^2^Department of Neurosurgery, Icahn School of Medicine at Mount Sinai, New York City, NY, USA.; ^3^Technical University of Munich, Munich, Germany.; ^4^Department of Neurosurgery, University of Alabama at Birmingham, Birmingham, AL, USA.; ^5^School of Medicine, University of Glasgow, Glasgow, UK.; ^6^Department of Neurosurgery, Duke University Medical Center, Durham, NC, USA.

**Keywords:** Global Neurosurgery, Traumatic Brain Injury, Surveillance, Neurotrauma, Trauma Registry, Health Systems

## Abstract

**Background:** Injury is a major global health problem, causing >5 800 000 deaths annually and widespread disability largely attributable to neurotrauma. 89% of trauma deaths occur in low- and middle-income countries (LMICs), however data on neurotrauma epidemiology in LMICs is lacking. In order to support neurotrauma surveillance efforts, we present a review and analysis of data dictionaries from national registries in LMICs.

**Methods:** We performed a scoping review to identify existing national trauma registries for all LMICs. Inclusion/ exclusion criteria included articles published since 1991 describing national registry neurotrauma data capture methods in LMICs. Data sources included PubMed and Google Scholar using the terms "trauma/neurotrauma registry" and country name. Resulting registries were analyzed for neurotrauma-specific data dictionaries. These findings were augmented by data from direct contact of neurotrauma organizations, health ministries, and key informants from a convenience sample. These data were then compared to the World Health Organization (WHO) minimum dataset for injury (MDI) from the international registry for trauma and emergency care (IRTEC).

**Results:** We identified 15 LMICs with 16 total national trauma registries tracking neurotrauma-specific data elements. Among these, Cameroon had the highest concordance with the MDI, followed by Colombia, Iran, Myanmar and Thailand. The MDI elements least often found in the data dictionaries included helmet use, and alcohol level. Data dictionaries differed significantly among LMICs. Common elements included Glasgow Coma Score, mechanism of injury, anatomical site of injury and injury severity scores. Limitations included low response rate in direct contact methods.

**Conclusion:** Significant heterogeneity was observed between the neurotrauma data dictionaries, as well as a spectrum of concordance or discordance with the MDI. Findings offer a contextually relevant menu of possible neurotrauma data elements that LMICs can consider tracking nationally to enhance neurotrauma surveillance and care systems. Standardization of nationwide neurotrauma data collection can facilitate international comparisons and bidirectional learning among healthcare governments.

## Introduction

 Traumatic injury is a poorly recognized global public health crisis, representing a major cause of morbidity and mortality in virtually every nation and demographic.^1^ Notably, 89% of trauma-related mortalities occur in low- and middle-income countries (LMICs),^2,3^ with the leading cause of trauma-related deaths worldwide being brain injury.^4^ Considering that inhabitants of LMICs are three times more likely to sustain a traumatic brain injury (TBI)^5,6^ and are much less likely to receive the standard of medical care offered to patients in high-income countries (HICs),^7^ populations in LMICs are at a disproportionately high risk for TBI-related death and disability. This disparate trauma-related risk also carries significant economic implications given that the young demographics affected by injuries often tend to be primary income earners for families.^6^

*Neurotrauma,* which is defined as traumatic injury to the head, brain, and/or spine, is therefore a public health concern also of great economic importance to nations of all income levels. Nationwide reduction of the neurotrauma burden in all countries requires public health actions that enable government officials to understand and monitor the local epidemiology of traumatic brain and spine injury. These actions, collectively referred to as “public health surveillance,” include the ongoing systematic collection, analysis and interpretation of data on neurotrauma, and a close integration of that data with its timely dissemination to government offices and ministries that are accountable for injury control and prevention.^8^ Recognizing the barriers to trauma surveillance in many LMICs, which contribute to an absence or inadequacy of routine data collection at the health facility level, this article focuses on national trauma registries as a prospective source of neurotrauma surveillance data in LMIC contexts. Combining a systematic scoping review of the literature on national trauma registries in LMICs with non-randomized sampling methods, we analyze the data dictionaries of national trauma registries from 14 LMICs on five different continents. By focusing our inquiry and analysis on LMICs, we aim to provide contextually relevant recommendations to other LMIC healthcare governments regarding the most useful neurotrauma data to systematically collect at the district hospital level.

## Methods

###  Search Strategy

 This study utilized two approaches aimed at understanding current frameworks for national neurotrauma data capture at the district hospital level in LMICs. First, a systematic scoping review of the literature was performed aimed at identifying LMICs with a published experience in national neurotrauma data collection using national trauma registries, and cataloging their neurotrauma data dictionaries.^9^ These results were then augmented with analysis of neurotrauma data dictionaries from an LMIC convenience sample, and “cold contact” sampling of LMIC ministries of health based on contact information available through their web sites. Each of these methods is described below.

###  Systematic Scoping Review: Selection Criteria

 The protocol for this review followed the guidelines of the Joanna Briggs Institute for scoping reviews.^9^Table 1 presents the inclusion and exclusion criteria for the review of the literature, which were defined to limit results to published reports of nationwide trauma registries, ie, not limited to a single hospital or an exclusive region of a country. International neurotrauma registries that encompassed several countries were also excluded. We included reports on LMIC national trauma registries published since 1991 that included neurotrauma-related elements, and were available in either English, French, Spanish or German.

**Table 1:** Overview of Inclusion and Exclusion Criteria

**Inclusion Criteria**	**Exclusion Criteria**
English/French/Spanish/German full-text PDF available.	No PDF available in one of the four included languages.
Published after 1/1/1991.	Published before 1/1/1991.
A LMIC according to World Bank online classification at time of literature search (October 2019).	Not an LMIC at time of search.
A national trauma registry.	Trauma registry for a single facility, a limited region of a country, or for multiple countries.
Registry must include neurotrauma-specific elements.	Registry elements are not specific to neurotrauma.
Article is primary research article.	Review article.
Article is a complete manuscript.	Conference abstract only.
Nation exists at time of research.	Nation inexistant at time of research.

Abbreviation: LMIC, low- and middle-income country.

**Figure 1:**
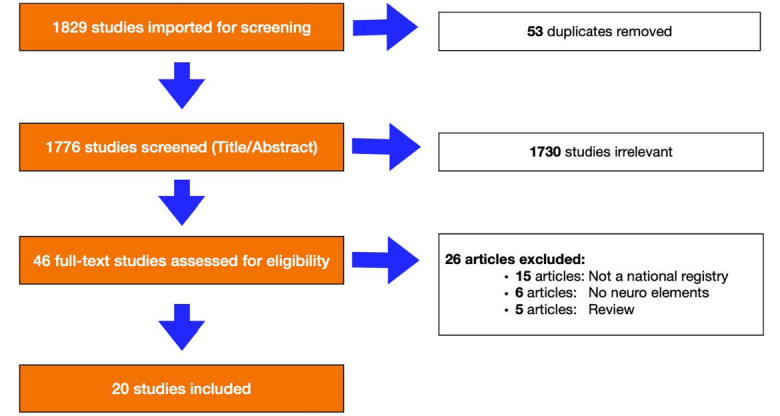
Preferred Reporting Items for Systematic Reviews and Meta-Analyses Diagram of Our Systematic Scoping Review.

Figure 1 illustrates our flow diagram, using Preferred Reporting Items for Systematic Reviews and Meta-Analyses. Utilizing a query designed to identify articles from LMICs that met our criteria, we performed a PubMed literature search on October 14, 2019 which resulted in 1776 articles following removal of duplicated results. Title and abstract screen were performed by five of our authors (AH, EJB, RB, EC and JA), with at least two authors screening each article for inclusion or exclusion. Following this first screening, 1730 articles were eliminated (Figure 1). Next, the same authors performed a full-text screen on the remaining 46 articles using the criteria outlined in Table 1. This process again required review by at least two authors, with one author (EJB) confirming the final selection of articles for extraction. This resulted in 20 articles meeting all protocol criteria. The neurotrauma data elements of LMIC national trauma registries from these articles were then extracted and cataloged for further analysis (see Results).

###  Non-randomized Sampling (Convenience and Cold Contact): Selection Criteria

**Figure 2:**
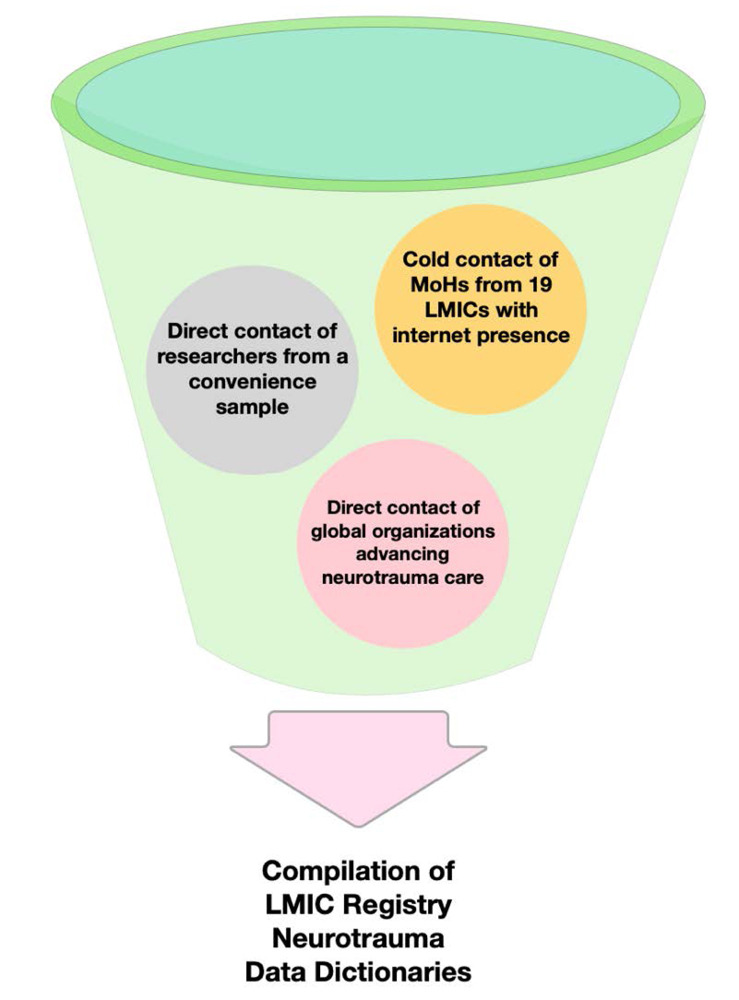
Diagrammatic Representation of the Non-randomized Sampling Methods Used to Compile Neurotrauma Data Dictionaries Used in National LMIC Trauma Registries. Abbreviations: MoHs, ministries of health; LMIC, low- and middle-income country.

 In order to augment the results of our literature review, we used three methods of non-randomized sampling: convenience sampling of researchers, cold contact with ministries of health and cold contact with global organizations that participate in advancing neurotrauma care. These methods are outlined in Figure 2. Convenience sampling involved contacting key informants known to conduct relevant global surgery policy research in LMICs, and requesting information on data dictionaries currently being used to track neurotrauma on a national scale. Then, we used a cold contact sampling method by performing an internet search for the contact information of ministries of health^10^ in various LMICs on five different continents (Africa, Asia, Oceania, Eastern Europe, Central America). Since not all Ministry of Health (MoH) in all LMICs worldwide could be contacted, 19 countries were randomly chosen based on the results of internet searches. These ministries were directly contacted to find out whether their surveillance programs included a national neurotrauma or trauma registry. Means of communication included directly sending email messages addressed to MoH officials, filling out online contact forms on official MoH websites, and attempting telephone contact whenever MoH telephone numbers were available. In the case of telephone contact, each MoH was called on at least three separate occasions during daytime work hours, accounting for time zone differences as necessary. Any resulting data was included in our cataloging and analysis (see results). Finally, using a similar cold contact sampling method, we contacted global organizations that are currently engaged in advancing TBI care for assistance in identifying LMICs with national trauma or TBI registries. These included the World Federation of Neurosurgical Societies, and the International Neurotrauma Society, and the World Health Organization (WHO). TBI registry data elements identified through these contacts were also cataloged and analyzed, with a goal to augment our final recommendations. Resulting data is reported below.

###  Comparison With Minimum Dataset for Injury 

 In order to assess and reconcile the comprehensiveness of data dictionaries from all identified registries against a global standard, the former were compared to the minimum dataset for injury (MDI) embedded in WHO’s International Registry for Trauma and Emergency Care (IRTEC). Results of this comparison appear in Table 2.

**Table 2:** Comparison of Data Elements From Included Studies and the WHO Minimum Dataset for Injury Variables

**MDI Data Element**	**Cameroon** ^a^	**Colombia** ^b^	**Iran ** **NSCIR-IR** ^20^	**Myanmar** ^5^	**Iran INTRD ** ^19,21-23^	**Thailand** ^37^	**Mexico** ^30^	**India** ^33^	**Pakistan ** ^28,36^	**Malaysia** ^26,34^	**Rwanda** ^29^	**Fiji ** ^16-18^	**Jamaica NTR** ^25^	**Egypt ** ^27^	**Jamaica ISS ** ^24^	**China NISS** ^13-15,32^
Admission vitals	P	P	P	P	P	P			P	P	P					
Anatomic location	P	P	P	P			P			P	P	P				P
AVPU score	P															
GCS	P	P	P	P	P	P		P	P	P	P		P			
Injury mechanism	P	P	P	P	P	P	P	P	P	P	P	P	P	P	P	P
Helmet/protective device	P		P	P	P	P	P	P							P	
Alcohol	P	P		P		P	P	P				P			P	
Method of arrival	P	P	P	P	P	P	P	P	P		P					
In hospital procedures	P	P	P		P		P	P	P	P			P	P		
Discharge disposition	P	P	P	P	P	P	P		P	P	P	P	P	P		P

Legend: P = the country corresponding to this column collects this data element. Grey filling = the country corresponding to this column does not collect this data element. Order of the countries: Those on the top left (Cameroon) collect most of the recommended data elements, on the bottom right the least (China NISS). ^a^Personal contact with Catherine Juillard after reading Juillard et al.^32^ ^b^Personal contact with Andres Rubiano. Abbreviations: WHO, World Health Organization; MDI, minimum dataset for injury; INTRD, Iran National Trauma Registry Database; NSCIR-IR, National Spinal Cord Injury Registry of Iran; NTR, National Trauma Registry; ISS, Injury Surveillance System; NISS, National Injury Surveillance System; GCS, Glasgow Coma Scale.

## Results

###  Systematic Scoping Review: Data Extraction

 The summary of our literature review appears in Figure 1. We found 20 studies reporting on 10 national trauma registries for Brazil,^11,12^ China,^13-15^ Fiji,^16-18^ Iran,^19-23^ Jamaica,^24,25^ Malaysia,^26^ Egypt,^27^ Pakistan,^28^ Rwanda^29^ and Mexico.^30^ The trauma registry data for Brazil was unobtainable, so we included 9 different countries in five different WHO regions: Africa (Rwanda), the Americas (Jamaica and Mexico), the Eastern Mediterranean (Egypt, Iran and Pakistan), South-East-Asia (India), the Western Pacific (China, Fiji and Malaysia). These countries also represented the full spectrum of World Bank income levels for LMICs, ranging from low- to upper- middle-income countries (see Supplementary file 1: Table S1).^14-37^ Among these studies, strengths of the data collection process included use of nternational Classification of Diseases (ICD)-9 codes, inclusion of elements that were specific to neurotrauma, use of a trauma severity score.

 Various members of hospital staff were entrusted with the data collection depending on the site, ranging from nursing students to physicians. In large Chinese hospitals, nurses filled in the registry forms whereas in smaller Chinese hospitals, physicians directly collected the data.^32^ Similar to large Chinese hospitals, Iranian registry forms were filled in by “registrars” in the case of NSCIR (National Spinal Cord Injury Registry of Iran)^20^ or by “trained physicians” in the case of INTRD (Iran National Trauma Registry Database).^19^ In Fiji, data was entered by “trainee interns”^18^ or “research assistants and hospital nurses.”^17^ In Rwanda, a publicly funded prehospital emergency system employed trained nurses and anesthetists to provide emergency care, and submit the data from each clinical encounter.^29^

 The tools for data collection and storage also varied significantly among sites. Countries relying on paper for data collection with subsequent computer entry included Cameroon,^31^ Pakistan,^36^ and India.^33^ In contrast electronic data collection and web-based storage was utilized by several national databases including Malaysia,^26^ Jamaica^24,25^ and the Iranian NSCIR,^20^ all of which used a different custom web-based application. In Rwanda, an electronic secure web-based prehospital registry using a REDCap database, developed at Vanderbilt University in Nashville, Tennessee, USA, had been previously created in collaboration with the authors of the study.^29^ The neurotrauma-specific data elements tracked by the national registries identified in this review are presented in Supplementary file 1 (see Table S1). In addition to items that specifically characterize TBI, such as Glasgow Coma Scale, anatomic site of injury, and availability/findings of head and brain injury studies, items such as substance use and use of safety devices are included for their relevance to neurotrauma care and outcomes.

###  Non-randomized Sampling: Data extraction

 Cold contact of ministries of health resulted in only one reply among the 19 contacted ministries: the Kenyan MoH. The trauma registry kept by the respondent is, however, regional and therefore did not meet our criteria for national trauma registries. Searching the websites for these health ministries generated comprehensive data dictionaries from India and Myanmar. They were freely available online through the websites of their respective official health ministry websites (see Table 2 and Table S1**)**. Key informants from our convenience sample included researchers working on the Colombian national TBI registry, and investigators with access to the National Trauma Registry of Cameroon as well as investigators from India’s Indian Registry of Intensive Care. These contacts provided the data dictionaries from their registries upon request. Data elements from Colombia and Cameroon are included in Table 2 and Table S1.Since literature review revealed more broadly defined national trauma registry data elements for India coming from the National Injury Surveillance Trauma Registry and Capacity Building Center (NISC) of India rather than from the Indian Registry of Intensive Care, data elements from the NISC were selected for Table 2 and Table S1.

 Direct contact with the World Federation of Neurosurgical Societies and the International Neurotrauma Society reinforced existing data from our convenience sample, such as data from the Colombian Neurotrauma Registry. No new data dictionaries resulted, however, from these communications. The “WHO 2009 Workshop on Injury Surveillance”^37^ provided further data from the national trauma surveillance systems of Thailand and Myanmar.

###  Quality Assessment: Comparison With Minimum Dataset for Injury 

 Among the 16 registries presented in Table 2, only Cameroon’s registry included all elements of the MDI. Five registries included all but one MDI element, while 9/15 registries included at least 6 of the 8 recommended data elements from the MDI.

## Data Analysis and Discussion

###  The Unmet Need for National Neurotrauma Registries in LMICs

 In the era of sustainable development, the global neurosurgery movement has highlighted extraordinary data asymmetry across nations by income level. In HICs, the use of registry-based data for prognostic modeling and improvements in neurotrauma care and outcomes based on these data-driven models, have been shown to comprise a key component of trauma quality assurance efforts.^38-40^ Such efforts continue to empower rich data collection and reporting initiatives in these countries, further widening the gap between them and LMICs; indeed, the neurotrauma data sparsity from LMICs is also well documented.^10^ Therefore, the need for developing effective neurotrauma surveillance systems in LMICs has emerged as a priority of global health policy.^41,42^ It is our supposition that improvement and standardization of neurotrauma data elements would save lives in LMICs and that neurosurgeons have a key role to play in informing this process. This premise is supported by the findings of Kesinger et al who found decreased mortality from brain and spine injury after the implementation of standardized protocols for neurotrauma data collection in LMICs.^43^

 The paucity of data on the neurotrauma epidemiology of LMICs is readily appreciated by findings from the WHO global burden of disease (GBD) study, where epidemiological studies, literature review, hospital-based reports and modeling-driven data generated estimates with significant geographic variations that were not easily explained.^10^ For instance, in the GBD of 2016, neurotrauma rates were lower in some LMIC regions such as sub-Saharan Africa, than in North America or Western Europe, where greater healthcare resource wealth may generate survival bias, and robust data collection systems provide input for modeled estimates that is lacking in many lower sociodemographic regions.^10^ As noted in the GBD report on neurotrauma, registry systems with data dictionaries for traumatic brain and spine injury could refine the accuracy of future assessments while simultaneously empowering further global neurotrauma research efforts.^10^

 In our study, extensive research has only led us to sixteen national trauma registries in LMICs. Certainly the cause of such data asymmetry is multifactorial^44,45^ including: inadequacy of specialized clinical workforce, equipment and infrastructural requirements for data collection, burdensome financial costs associated with registry development and maintenance, lack of adequate healthcare policy implementation, and overwhelming clinical volume.^45^ Rubiano et al note that comprehensive trauma registries operate in only 29 of 115 countries that report such statistics to the WHO.^46^ These limitations underlie a chronically, yet critically unmet need for national data collection on TBI in LMICs. While such obstacles will likely require very long-term solutions in LMICs we propose that there are also practical short-term solutions that could improve the quantity and quality of neurotrauma data collection in LMICs.

###  Key Elements to Guide Registry Data Capture

 In 2019, the WHO released the IRTEC, which is a free web-based platform for global collection of trauma data. It utilizes a validated MDI^47^ to collect practical clinical information at the patient level.^48,49^ Users can download paper data collection forms or directly input the data electronically for later analysis and quality improvement.^50^ In an ideal setting, comprehensive surveillance of head, brain and spine injuries would include aggregation of data from hospital records, death certificates, coroner records, and emergency medical service records. However, in the earliest stages, standardized clinical data collection from multiple busy trauma centers throughout the country into a single database is an ideal starting point.

 Comparison of the databases found in our search with the WHO MDI demonstrated several points of commonality but also important deviation that left significant gaps in data collection. Countries such as Jamaica and China, demonstrating a minimum of correspondence to the WHO MDI, may stand to improve upon existing data collection systems by including the missing MDI elements in their trauma registries. Moreover, a collective strategy of standardizing registry data collection across countries, may facilitate international comparisons and bidirectional learning among healthcare governments.

 Notably, we found that several countries utilized paper collection tools while many others implemented electronic platforms. Additionally, in each country the actual data collection was performed by people with varying medical training backgrounds. We propose that the modality of collection should be context-specific based on the capability and resources of the individual facility and trauma provider. The most important factor is determining choice of data collection that will allow the greatest amount of patient inclusion and data accuracy. To this end, quality assessment and data validation are important components to any clinical registry. Two tools that can be utilized for this include the WHO Trauma System Maturity Index^51^ and the Evaluation Framework for Injury Surveillance Systems.^52^ In Uganda, the Kampala internet-based TBI Registry was designed in a way that made it possible to be evaluated by the Evaluation Framework for Injury Surveillance Systems.^53^

 The potential benefits of employing common data standards are extensive. Such efforts would facilitate data sharing between countries and localities and research collaboration. Quantitative analysis of patient outcomes would allow identification of trends in mortality and morbidity that could lead to quality improvement measures. Additionally, current evidence-based guidelines in trauma and neurocritical care are based nearly entirely on data arising from HICs. As such, it is possible that the guidelines may have limited applicability in limited resource settings, which are the setting for the greatest proportion of traumatic brain and spine injuries worldwide. Greater representation of high quality LMIC trauma data could serve to improve this inequity. In order to strengthen policies for traumatic injuries of the brain and spine, ministries of health should work with local neurosurgeons to prioritize the development of local standards for neurotrauma case definitions and data dictionaries.^54,55^ This standardization could be achieved by adopting international guidelines and using global platforms such as the WHO IRTEC and MDI, while also employing context specific data collection modalities based on local resources and needs.

###  Limitations

 We acknowledge several limitations of this work. First, our literature review was limited in its ability to identify current trauma registries used in LMICs as authors from those countries or collaborating with investigators in those countries would need to have published their data dictionaries in the international peer-reviewed literature in order for them to be searchable by this method. Second, a lower response rate than desired in our non-random sampling methods limited our sample size of LMIC MoH, as well as our ability to identify whether contacted nations may store data outside of ministries of health, such as through outsourced third parties. Finally, our study was designed to identify neurotrauma-related data elements currently in use in LMICs, and therefore is limited in its ability to report the impact of these data dictionaries on the neurotrauma burden in the countries under study. As more LMICs develop their neurotrauma surveillance capacity and methods, the impact of these government-level actions on national neurotrauma burden presents an important area of public health and health policy research to guide continued efforts in global neurotrauma care.

## Conclusion

 The global burden of neurotrauma affects all countries, yet a disproportionately large percentage of that burden impacts LMICs. Trauma registries are either underutilized, or non-existent in most LMICs despite the acknowledged need for these facility-level data platforms. The absence of this facility-level data translates into an equivalent absence of national data. We recommend the use of nationally standardized trauma registries using the WHO MDI and IRTEC datasets with neurotrauma-specific data elements as a key source of surveillance data for ministries of health.^44,51^ The data dictionaries presented in this report may serve as a guide to other LMICs on prospective national neurotrauma registry design, as well as an opportunity for reported LMICs to continue improving upon existing data collection methods.

## Acknowledgements

 This paper has not been presented at any meetings. Abstracts reporting on the progress of this work were presented as an electronic poster at the 2019 Annual Scientific Meeting of the American Association of Neurological Surgeons, and as an oral presentation at the 2020 Rutgers University Virtual Meeting called, “Global Neurosurgery: Ask Not for Whom the Bell Tolls.”

## Ethical issues

 Not applicable.

## Competing interests

 Authors declare that they have no competing interests.

## Authors’ contributions

 EJB conceived and designed this work, acquired, analyzed and interpreted the data, drafted and revised the manuscript, corresponded with reviewers, carried out the revision process, and prepared the final version to be published. AECH co-designed this work, acquired, analyzed and interpreted the data, co-drafted and revised the manuscript, participated in revision and approved the final version to be published. JL critically reviewed the manuscript, edited and co-drafted the work, and approved the final version to be published. JA co-designed this work, analyzed the data, and approved the final version of the manuscript. RBB acquired and analyzed the data, co-drafted the manuscript and approved the final version of the manuscript. EC acquired and analyzed the data, co-drafted the manuscript and approved the final version of the manuscript. JC critically reviewed the manuscript, edited the work and approved the final version to be published. KBP co-conceived and co-designed this work, supervised project development, critically reviewed the manuscript and approved the final version to be published.

## Funding

 No grant funding was received for completion of this study. During this study, Anna E Hackenberg received scholarship support from *Studienstiftung des deutschen Volkes*, the German Academic Scholarship Foundation.

## Supplementary files

Supplementary file 1 contains Table S1 and search query used for literature review.
