# Supplementary material for: Neurotrauma Surveillance in National Registries of Low- and Middle-Income Countries: A Scoping Review and Comparative Analysis of Data Dictionaries
Source: Int J Health Policy Manag. 2021 Dec 6;11(11):2373–80. doi: 10.34172/ijhpm.2021.167 (PMC9818108; doi:10.34172/ijhpm.2021.167)
Supplement: Supplementary file 1 — contains Table S1 and search query used for literature review. [file ijhpm-11-2373-s001.pdf]

Article title: Neurotrauma Surveillance in National Registries of Lowand Middle-Income Countries: A Scoping Review and Comparative Analysis of Data Dictionaries

Journal name: International Journal of Health Policy and Management (IJHPM)

Authors' information: Ernest J. Barthélemy[1, 2]*¶, Anna E. C. Hackenberg[1,3]¶, Jacob Lepard[1,4], Joanna Ashby[1,5], Rebecca B. Baron[2], Ella Cohen[2], Jacquelyn Corley[1,6], Kee B. Park[1]

[1]Program in Global Surgery and Social Change, Department of Global Health and Social Medicine, Harvard Medical School, Boston, MA, USA.
[2]Department of Neurosurgery, Icahn School of Medicine at Mount Sinai, New York City, NY, USA.
[3]Technical University of Munich, Munich, Germany.
[4]Department of Neurosurgery, University of Alabama at Birmingham, Birmingham, AL, USA.
[5]School of Medicine, University of Glasgow, Glasgow, UK.
[6]Department of Neurosurgery, Duke University Medical Center, Durham, NC, USA

(*Corresponding author: globalneurosurgeon@gmail.com)
¶Both authors contributed equally to this paper

Supplementary file 1

Table S1. Summary of Neurotrauma-Specific Data Elements From All Studies Included After Systematic Scoping Review and Non-Random Sampling

**Country, WHO Region**	**World Bank Income Status (2020)**	**Neurotrauma Specific Data Elements**
Cameroon, Africa[31]<br>Personal contact with Catherine Juillard	Lower Middle income country (L-MIC)	AIS: Head & Neck (1-5);<br>AVPU score, GCS/AVPU qualifier;<br>C-collar in place?;<br>Complications: Seizures (the day of admission to hospital, the day after surgery: yes/no), seizure treatment given in form of medicine? No seizure treatment: Why?;<br>ER Head CT;<br>Follow-up: Head/Neck- trauma,<br>GCS, GOS at discharge;<br>Head/Neck severity<br>ICU: Anti-seizure prophylaxis, Seizures (If yes, how many hours?);<br>ICU: C-Spine X rays; ICU: Head CT; ICU: C-Spine CT;

		ICU: Neuromonitoring;<br>If applicable: Use of Helmet;<br>If head injury then GCS;<br>Injured Part of the Body: Head, Spine;<br>Observation of Head/CNS;<br>Pupil reactivity at ER;<br>Received pre-hospital care: c-spine immobilization;<br>Received pre-hospital-care: Back Board;<br>Surgical re-intervention;<br>Surgical treatment received;<br>Survival Status: GOS at 3, 6, 12 months;<br>Type of Surgery Performed
China NISS, Western Pacific [14, 15, 32]	Upper-middle-income-country (UMIC)	Anatomic site of injury including head, neck, TBI;<br>“Body part injured: nervous system”;<br>Disposition/Outcome; Mechanism / cause of injury<br>Injury severity (ISS, minor/ moderate/severe)
Colombia, Americas (personal contact with Andres Rubiano)	UMIC	AIS: Head & Neck (1-5);<br>Discharge AIS: Head and Neck (1-5);<br>ER Head CT; C-Spine CT;<br>GCS (if head injury);<br>GCS, GOS at discharge;<br>ICU: Anti-seizure prophylaxis, Seizures (If yes, how many hours?);<br>ICU: C-Spine CT;<br>ICU: C-Spine X rays;<br>ICU: Head CT;<br>ICU: Minimum, Maximum GCS of day;<br>ICU: Neuromonitoring;<br>ICU: Pupil Reactivity (For Right and Left Pupils);<br>Injured Part of the Body: Head, Spine;<br>Observation of Head/CNS;<br>Pre-hospital GCS;<br>Pupil reactivity at ER;<br>Surgical re-intervention;<br>Survival Status: GOS at 3, 6, 12 months
Egypt, Eastern Mediterranean[27]	L-MIC	Mechanism / cause of injury
Fiji, Western Pacific [16-18]	UMIC	Anatomic site of injury including head, neck, TBI;<br>Disposition/Outcome;

		Injury severity (ISS, minor/moderate/severe);<br>Mechanism/cause of injury<br>Substance Use
India, South-East-Asia [33]	L-MIC	AIS: Head & Neck (1-5);<br>ER Head CT;<br>GCS (if head injury);<br>History of consciousness;<br>If applicable: Use of Helmet;<br>Injured Part of the Body: Head, Spine;<br>Surgical treatment received;<br>Type of Surgery Performed
Iran INTRD, Eastern Mediterranean [19, 21-23]	UMIC	Disposition /Outcome;<br>Glasgow Coma Scale;<br>ICD-9 or 10 Code;<br>Injury severity (ISS, minor/moderate/severe);<br>Mechanism/cause of injury<br>Spinal Injury score/characteristics* (ASIA, etc);<br>Spine injury;<br>Use of helmet;<br>Use of seatbelt
Iran NSCIR-IR, Eastern Mediterranean[20]		"Body part injured: nervous system";<br>Anatomic site of injury including head, neck, TBI;<br>Complications after treatment of spine injury;<br>CSF fluid out of the wound;<br>Disposition/Outcome;<br>Glasgow Coma Scale;<br>ICD-9 or 10 Code;<br>Mechanism / cause of injury<br>Mentions neurosurgery department or intracranial surgery;<br>Spinal Injury score/characteristics* (ASIA, etc);<br>Spinal surgery;<br>Spine injury;<br>Use of helmet;<br>Use of seatbelt;
Jamaica ISS, Americas[24]	UMIC	Mechanism / cause of injury;<br>Substance use;<br>Use of helmet

Jamaica NTR,<br>Americas[25]		Injury severity (ISS, minor/moderate/severe);<br>Glasgow Coma Scale;<br>Glasgow Outcome Scale at discharge;<br>Head/Brain imaging;<br>Mechanism/cause of injury
Malaysia, Western Pacific[26, 34]	UMIC	Anatomic site of injury including head, neck, TBI;<br>Disposition/Outcome;<br>Glasgow Coma Scale;<br>Head/Brain imaging; Spinal surgery;<br>Injury severity (ISS, minor/moderate/severe);<br>Mechanism/cause of injury;<br>Mentions neurosurgery department or intracranial surgery;<br>Spinal Injury score/characteristics* (ASIA, etc);<br>Urgent surgery (< 24h);
Mexico[30]	UMIC	Affected anatomy: head, neck, spinal column, back and gluteal region;<br>Major sequelae: contusion, fracture, “depression” = (mechanical) depression, scars;<br>Protective equipment: helmet, seatbelt
Myanmar, South-East-Asia[35]	L-MIC	History of consciousness;<br>If applicable: Use of Helmet;<br>Injured Part of the Body: Head, Spine;<br>Outcome
Pakistan, Eastern Mediterranean[28 36]	L-MIC	Disposition/Outcome;<br>Glasgow Coma Scale;<br>Head/Brain imaging;<br>Mechanism/cause of injury
Rwanda, Africa[29]	Low<br>income<br>country	Anatomic site of injury including head, neck, TBI;<br>Disposition/Outcome;<br>Glasgow Coma Scale;<br>Injury severity (ISS, minor/moderate/ severe);<br>Mechanism / cause of injury;<br>Spine injury
Thailand, South-East-Asia[37]	UMIC	History of consciousness;<br>If applicable: Use of Helmet;<br>Injured Part of the Body: Head, Spine;<br>Observation of Head/CNS;<br>Outcome

Search Query Used for Literature Review

(country*[tiab] OR nation*[tiab] OR sovereign[tiab] OR royal[tiab] OR imperial[tiab] OR public[tiab] OR state[tiab] OR civil[tiab])AND(trauma*[tw] OR injur*[tw]) AND (regist*[tiab] OR databank[tiab] OR database*[tiab]) AND ("developing countries"[mesh] or developing countr*[tiab] or developing nation*[tiab] or less developed countr*[tiab] or less developed nation*[tiab] or third world nation*[tiab] or third world countr*[tiab] or under developed nation*[tiab] or underdeveloped nation*[tiab] or under developed countr*[tiab] or underdeveloped nation*[tiab] or middle income countr*[tiab] or middle income nation*[tiab] or low income countr*[tiab] or low income nation*[tiab] or poor countr*[tiab] or poor nation*[tiab] or lmic[tiab] or lmics[tiab] or "africa"[mesh] or "asia"[mesh] or "south america"[mesh] or "latin america"[mesh] or "central america"[mesh] or africa[tiab] or asia[tiab] or south america*[tiab] or latin america*[tiab] or central america*[tiab] or afghanistan*[tiab] or albania*[tiab] or algeria*[tiab] or samoa*[tiab] or angola*[tiab] or armenia*[tiab] or argentin*[tiab] or azerbaijan*[tiab] or bangladesh*[tiab] or bengali[tiab] or belarus*[tiab] or belize[tiab] or benin[tiab] or bhutan*[tiab] or bolivia*[tiab] or bosnia*[tiab] or herzegovina*[tiab] or botswana*[tiab] or brazil*[tiab] or bulgaria*[tiab] or burkina faso[tiab] or burundi*[tiab] or cabo verd*[tiab] or cape verd*[tiab] or cambodia*[tiab] or cameroon*[tiab] or central african*[tiab] or chad*[tiab] or china[tiab] or chinese[tiab] or colombia*[tiab] or comoros[tiab] or congo[tiab] or costa rica*[tiab] or cote d ivoire[tiab] or ivory coast[tiab] or croatia*[tiab] or cuba[tiab] or cuban[tiab] or djibouti[tiab] or dominica*[tiab] or ecuador*[tiab] or egypt*[tiab] or el salvador*[tiab] or eritrea*[tiab] or ethiopia*[tiab] or fiji*[tiab] or gabon*[tiab] or gambia*[tiab] or georgia*[tiab] or ghana*[tiab] or grenada*[tiab] or guatemala*[tiab] or guinea*[tiab] or guyan*[tiab] or haiti*[tiab] or hondura*[tiab] or india[tiab] or indian*[tiab] or indonesia*[tiab] or iran*[tiab] or iraq*[tiab] or jamaica*[tiab] or jordan[tiab] or kazakh*[tiab] or kenya*[tiab] or kiribati[tiab] or democratic people's republic of korea[tiab] or north korea[tiab] or kosovo[tiab] or kosovar*[tiab] or kyrgyz*[tiab] or lao[tiab] or laos[tiab] or laotian*[tiab] or lebanon[tiab] or lebanes*[tiab] or lesotho[tiab] or liberia*[tiab] or libya*[tiab] or macedonia*[tiab] or madagascar*[tiab] or malawi*[tiab] or malaysia*[tiab] or maldives[tiab] or mali[tiab] or malian[tiab] or marshall island*[tiab] or mauritania*[tiab] or mauriti*[tiab] or mexico[tiab] or mexican*[tiab] or micronesia*[tiab] or moldova*[tiab] or mongolia*[tiab] or montenegr*[tiab] or morocc*[tiab] or mozambique[tiab] or myanmar[tiab] or burmese*[tiab] or burma[tiab] or namibia*[tiab] or nauru*[tiab] or nepal*[tiab] or nicaragua*[tiab] or niger*[tiab] or pakistan*[tiab] or palau[tiab] or panama*[tiab] or paraguay*[tiab] or peru*[tiab] or philippin*[tiab] or romania*[tiab] or rwanda*[tiab] or russia*[tiab] or samoa*[tiab] or sao tome[tiab] or principe[tiab] or senegal*[tiab] or serbia*[tiab] or sierra leone*[tiab] or solomon island*[tiab] or somalia*[tiab] or south africa*[tiab] or sri lanka[tiab] or st lucia[tiab] or saint lucia[tiab] or st vincent[tiab] or saint vincent[tiab] or grenadines[tiab] or sudan*[tiab] or suriname*[tiab] or swaziland*[tiab] or syria*[tiab] or tajik*[tiab] or tanzania*[tiab] or thai[tiab] or thailand[tiab] or timor*[tiab] or togo*[tiab] or tonga*[tiab] or tunisia*[tiab] or turkey[tiab] or turkish[tiab] or turkmen*[tiab] or tuvalu*[tiab] or uganda*[tiab] or ukrain*[tiab] or uzbeki*[tiab] or vanuatu*[tiab] or venezuela*[tiab] or vietnam*[tiab] or viet nam*[tiab] or west bank[tiab] or gaza*[tiab] or palestin*[tiab] or yemen*[tiab] or zambia*[tiab] or zimbabw*[tiab])
